# Comprehensive Classification and Regression Modeling of Wine Samples Using ^1^H NMR Spectra

**DOI:** 10.3390/foods10010064

**Published:** 2020-12-30

**Authors:** Gábor Barátossy, Mária Berinkeiné Donkó, Helga Csikorné Vásárhelyi, Károly Héberger, Anita Rácz

**Affiliations:** 1 National Food Chain Safety Office, Directorate of Oenology and Alcoholic Beverages, Budaörsi út 141-145, H-1118 Budapest, Hungary; BaratossyG@nebih.gov.hu (G.B.); BerinkeineM@nebih.gov.hu (M.B.D.); CsikorneH@nebih.gov.hu (H.C.V.); 2 Department of Plasma Chemistry, Institute of Materials and Environmental Chemistry, Research Centre for Natural Sciences, Magyar Tudósok krt. 2, H-1117 Budapest, Hungary; heberger.karoly@ttk.hu

**Keywords:** wine, machine learning, spectroscopy, cross-validation, metabolomics

## Abstract

Recently, ^1^H NMR (nuclear magnetic resonance) spectroscopy was presented as a viable option for the quality assurance of foods and beverages, such as wine products. Here, a complex chemometric analysis of red and white wine samples was carried out based on their ^1^H NMR spectra. Extreme gradient boosting (XGBoost) machine learning algorithm was applied for the wine variety classification with an iterative double cross-validation loop, developed during the present work. In the case of red wines, Cabernet Franc, Merlot and Blue Frankish samples were successfully classified. Three very common white wine varieties were selected and classified: Chardonnay, Sauvignon Blanc and Riesling. The models were robust and were validated against overfitting with iterative randomization tests. Moreover, four novel partial least-squares (PLS) regression models were constructed to predict the major quantitative parameters of the wines: density, total alcohol, total sugar and total SO_2_ concentrations. All the models performed successfully, with *R*^2^ values above 0.80 in almost every case, providing additional information about the wine samples for the quality control of the products. ^1^H NMR spectra combined with chemometric modeling can be a good and reliable candidate for the replacement of the time-consuming traditional standards, not just in wine analysis, but also in other aspects of food science.

## 1. Introduction

As Hungary is one of the top ten wine producing countries in Europe with twenty-two different wine regions, the quality control of Hungarian wines has utmost importance [1].

Wine is a complex beverage with numerous metabolites and other compounds that can characterize the products. It is well-known that the amount of different metabolites and the chemical profile of the wines depend on the environmental parameters of the region (soil, temperature, wine variety *etc*.) [2,3]. In the past few decades, several analytical techniques were developed for complex wine analysis, such as chromatographic and spectroscopic methods. Nuclear magnetic resonance spectroscopy (NMR) (sometimes coupled with isotope analysis) is one of the best ones for this task because of the high sensitivity for the chemical characterization of the wine samples [4,5]. NMR spectroscopy became a common tool for metabolic analysis of wine samples in the recent years [6,7]. Classification studies based on ^1^H NMR spectra were dedicated even to the aging periods of the wines [8].

Some of the recent classification studies based on NMR spectroscopy are summarized in Table 1, including ^13^C and SNIF-NMR (Site-Specific Natural Isotope Fractionation) examples, as well. It should be mentioned that in most cases, NMR-related classifications employ different multivariate chemometric methods, such as principal component analysis (PCA), linear discriminant analysis (LDA) or partial least-squares discriminant analysis (PLS-DA) [9,10]. As machine learning algorithms have become an integral part of food analysis (foodomics), these tools are highly recommended for the quality control of wine samples, as well [11]. Interestingly, quantitative NMR-based wine analysis is less common in the literature than classification. There are only a few examples, mainly focusing on the compounds involved in the fermentation process, or minor metabolites [12,13]. However, these models can also contribute to the quality control of the wines (even in adulteration and fraud detection). Moreover, with the measurement of qualitative and quantitative parameters together, NMR can provide a comprehensive evaluation of the samples in a faster and easier way with the same precision and sensitivity compared to classical experimental tools such as HPLC or UV-Vis spectroscopy-based measurements. Chemometric analysis can be easily implemented in any NMR-based protocol for laboratories with continuous wine analysis.

Table 1 clearly shows that even though machine learning tools are widespread in food science, in this specific task they are underrepresented. Moreover, the number of samples is sometimes very small for a robust modeling, especially for prediction of the physical properties or metabolic concentrations.

In our study, we present the complex chemometric analysis of a total of 403 wine samples. Our aim was to examine the variety-based classification possibilities in the case of white and red wine samples with a machine learning algorithm. Moreover, we provide robust regression models for major and minor constituents and physicochemical parameters of the wines such as (i) total alcohol concentration, (ii) density, (iii) total sugar concentration and (iv) total SO_2_ concentration. This way, we will showcase the extended use of NMR spectroscopy in the whole process of wine analysis.

## 2. Materials and Methods

### 2.1. Samples

The National Food Chain Safety Office (Nébih/NFCSO, Hungary) inspects all the commercially available wine products in Hungary, and verifies their authenticity, and the major quality and quantity parameters. The samples in the study were coming from this activity. In total, 403 authentic wine samples were measured and evaluated based on different aspects. Sample sets for modeling were compiled based on the availability of the reference values. The number of the applied samples are summarized in Table 2.

The samples were diluted with 10% buffer in D_2_O with 3-(trimethylsilyl)propanoic acid sodium salt (TSP) as an internal standard as pretreatment for the NMR measurements. The pH of the samples was adjusted to 3.10. Finally, 600 µL of the sample solutions were transferred to a 5 mm NMR tube.

### 2.2. Reference Measurements

Reference measurements for the four regression models were performed based on OIV standards for wine analysis. Density measurements were performed at 20 °C by laboratory density meter (based on oscillation) (OIV-MA-AS2-01A:R2012). The total alcohol concentration was determined by an Alcolyzer (Anton Paar, Graz, Austria) bench-top device based on a NIR measurement between 1150 and 1200 nm. The detection range was between 0 and 30 *v*/*v*%. The total sugar concentration was measured by an HPLC standard method (OIV-MA-AS311-03: R2016). The total SO_2_ concentration was measured with an UV-Vis spectrophotometer (at 560 nm) after dialyzation through a gas-membrane to separate the interfering compounds from the sample matrix (OIV.FV.823). The equipment and reagents are summarized in detail in the abovementioned standard protocols.

### 2.3. ^1^H NMR Analysis and Spectral Preprocessing

^1^H NMR measurements were performed on a Bruker AVANCE III HD 400 MHz NMR Spectrometer FoodScreener™ system (Bruker, Biospin, Rheinstetten, Germany) equipped with a 5 mm PA BBI 400S1 H-BB-D-05 Z probe-head, operating at 9.4 T and observing ^1^H at 400.15 MHz. A BTpH combined-pH titration unit was also added to the equipment. The measurements were carried out at a temperature of 300 K. Water suppression with an additional suppression of ethanol were applied. The sweep width (SW) was 20.5 ppm. The spectra were aligned to the TSP signal (δ = 0 ppm). Exponential weighting function was used (0.3 Hz line broadening) before Fourier transformation with Topspin software 3.5 (Bruker, Biospin, Rheinstetten, Germany). The suppression of ethanol and water peaks was implemented in the pulse sequence (shown in Supplementary Materials Figure S1). The NMR measurements were based on the work of Godelman et al. [22].

Spectral intensities were normalized to total intensity. The spectral preprocessing was handled by Mestrenova 9.0 software (Mestrelab research, Santiago de Compostela, Spain). The bucketing [15] of the spectra was carried out with 0.01 width regions between δ 0.5 and 9.99 ppm. The regions of ethanol (δ 0.98–1.35 ppm and δ 3.43–3.85 ppm) and water (δ 4.78–4.86 ppm) satellites were excluded from the spectra. After the exclusion of these regions, the final dataset contained 860 variables (the bucketed regions) with the scaled spectral intensity values in the cells and the samples in the rows. A representative spectrum (after preprocessing) of a wine sample is shown in Supplementary material Figure S2.

### 2.4. Chemometric Analysis

Extreme gradient boosting (XGBoost) algorithm was applied for the classification of the wine varieties [23]. XGBoost is a very popular machine learning algorithm in every field of data science. The method originated from the work of Friedman and his coworkers [24,25]. Generally, tree-based ensemble methods combine more tree models to provide a final, more effective model with better predictive performances; XGBoost is based on this idea as well. The basis of the method is the boosting of the trees, where the sequentially built tree-based models are boosting the high-performance models amongst them with the minimization of the errors. In gradient boosting, the minimization process is based on a gradient descent algorithm. XGBoost is an optimized version, where tree-pruning, parallelized tree building, missing value handling and optimization to avoid overfitting are all included in the algorithm.

Classification modeling was carried out in the KNIME analytics platform (KNIME AG, 4.0.2, Zurich, Switzerland). An iterative double cross-validation workflow procedure with internal and external test sets and fivefold randomized cross-validation (CV) (with stratified class ratios) was developed for the effective validation of the models [26]. The performance of the models was expressed by the accuracy and the weighted area under ROC (receiver operating characteristic) curve values (AUC) [27]. The weightings were implemented according to the number of samples in each class. The developed classification workflow in KNIME is shown in Supplementary material Figure S3.

A traditional multivariate regression method, partial least squares regression (PLSR) was applied in regression analysis [28]. The idea of PLS combines multilinear regression (MLR) and principal component regression (PCR). Latent variables are calculated as the linear combination of the original X variables and the Y dependent (target) variable. The number of latent variables (PLS components) was selected based on two criteria: the first local minimum of the root mean squared error of cross-validation (RMSECV), or if it was not located, additional components were chosen only if the RMSECV value was improved (decreased) by at least 2%. The number of components was maximized to twenty. Randomized fivefold cross-validation (CV) with twenty iterations was used for internal validation, and a separate test set (based on a chronological order of the samples) was selected for each task before model building. MATLAB software (R2019a, Natick, MA, USA) with PLS toolbox (Eigenvector research, Inc., Manson, WA, USA) was applied for regression model building.

Genetic algorithm was used for variable selection both for classification and regression [29] to increase the predictive power of the models and to eliminate the possibility of overfitting. Moreover, permutation test (randomization test) was applied with 50 iterations for the verification of the models against overfitting. Outlier selection was applied for regression, where samples outside the 95% confidence interval on the scatterplot of the first two latent variables were excluded from further analysis.

## 3. Results and Discussion

### 3.1. Classification Results

**Figure 1:**
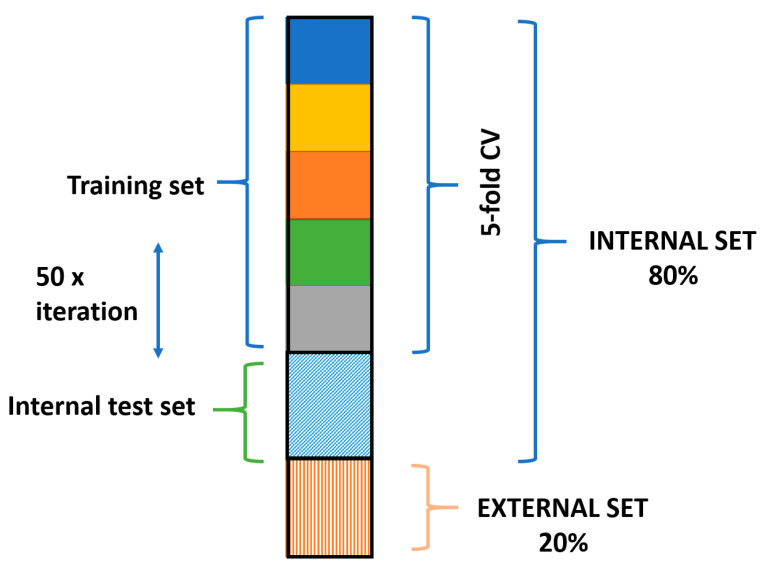
Summary of the dataset splits for modeling.

Three wine varieties were applied in white wine classification, namely Chardonnay, Sauvignon Blanc and Riesling, which were the most frequent groups amongst the samples. In total, 128 samples were used for modeling. The dataset was split into internal (training) and external (test) sets with an 80% split ratio and stratified sampling (preserving the ratio of samples in the three groups in the internal and external sets as well). The training set contained 102 samples. These samples were used in an iterative modeling workflow, where the internal set was randomly selected in 50 iterations. This has resulted in 50 models, and the mean of the predicted probability values was calculated for the final prediction of the class memberships. This protocol is based on a commonly referred protocol, called double cross-validation [26]. In the internal set, 70% of the samples were used for training (calibration) and for a fivefold randomized cross-validation (also with stratified sampling). The remaining 30% was used for internal test predictions. The workflow is summarized in Figure 1. The original variables were selected by genetic algorithm, and the final number of the used variables (buckets) was 84.

**Figure 2:**
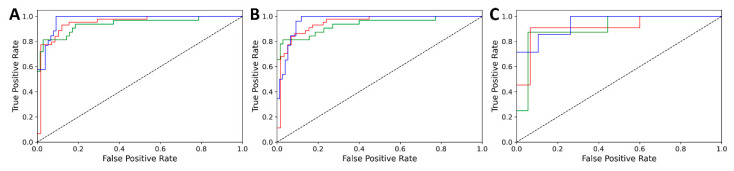
Three-class receiver operating characteristic (ROC) curves of the classification of white wine varieties, for cross-validation (**A**), internal test set (**B**) and external test set (**C**). Line colors are red for Riesling, blue for Sauvignon Blanc and green for Chardonnay.

The performance of the extreme gradient boosting (XGBoost) modeling for three white wine varieties was determined in terms of the accuracy and weighted-AUC (area under curve) values for the cross-validation (CV), internal test and external test set. The results are summarized in Table 3 and the respective plots are presented in Figure 2. The randomization (X-scrambling) test with 50 iterations showed that our final model was not overfitted.

**Figure 3:**
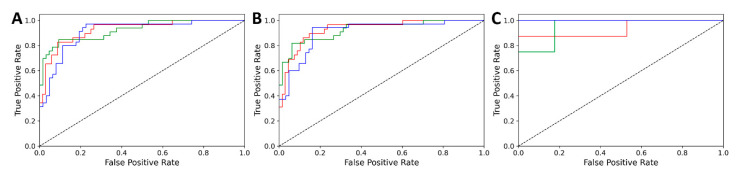
Three-class ROC curves of the classification of white wine varieties, for cross-validation (**A**), internal test set (**B**) and external test set (**C**). Line colors are red for Blue Frankish, blue for Cabernet Franc and green for Merlot.

The same procedure was applied for the three red wine varieties as well, namely: Cabernet Franc, Merlot and Blue Frankish. These three groups were the most populated amongst the used samples. Seventy-nine variables were selected from the original 860 with a genetic algorithm for XGBoost modeling. The performance parameters of the final model and the ROC curves of the validation set are summarized in Table 3 and Figure 3.

For both models, the accuracies were above 0.8 in each validation case, and the AUC values were above 0.9. These results are especially convincing for the external set, where the samples were entirely left out from the whole modeling phase. Figure 2 and Figure 3 show the *n*-class (three class) ROC curves, which highlight the consistently good results for each class and validation set: in fact, all of the curves are close to the hypothetical perfect classification (ROC curve going through the [0,1] point).

### 3.2. PLS Regression Results

Four models were built with PLS regression for (i) the density of the wine samples, (ii) the total alcohol content of the samples, (iii) the concentration of sugar and (iv) the total SO_2_ content of the wines. A genetic algorithm was applied to significantly reduce the number of variables and increase the robustness and reliability. Validation consisted of three parts: first, randomized fivefold cross-validation with 20 iterations was applied, then external test samples were used to verify the prediction performance of the model and finally, a permutation test with 50 iterations was applied to screen out the overfitted models. We have to note that not all of the 403 samples were measured with all four reference measurements, thus a subset of them was used for each model.

**Figure 4:**
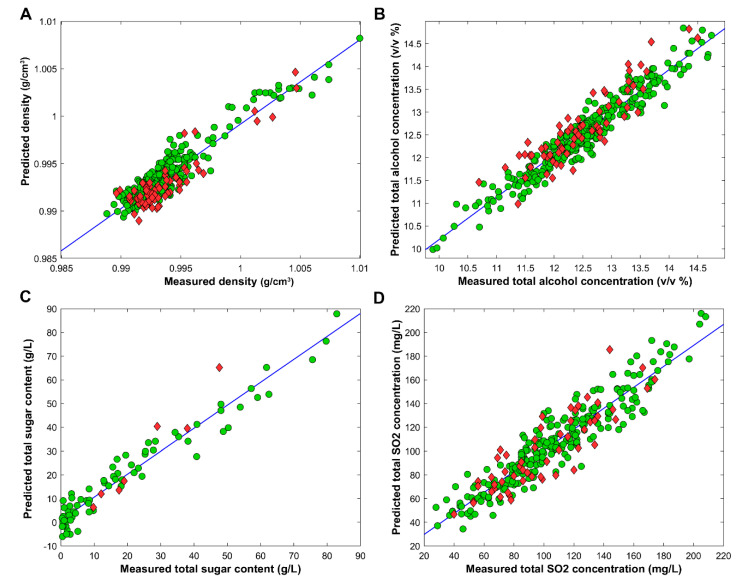
Measured and predicted values are plotted against each other for (**A**) density, (**B**) total alcohol concentration, (**C**) total sugar content and (**D**) total SO_2_ concentration. Training samples are marked with green circles, external samples are marked with red diamonds. The calibration curve is marked with a blue line.

In the case of density prediction, 260 variables were selected for modeling by genetic algorithm. The training set contained 278 samples and the external test set contained 71 samples. The external set was put aside prior to modeling; the samples were excluded based on their exact chronological order. Six latent variables were sufficient for PLS modeling (based on the global minimum of the RMSECV values). The performance of the model was calculated for the training (calibration), the CV and the external test set. *R*^2^ values were 0.88 (training), 0.86 (CV) and 0.82 (external validation). The permutation test with 50 iterations verified that the model was significantly different compared to the use of random numbers. The root mean squared error of the training, CV and external test set were 0.0011, 0.0012 and 0.0015 g/cm^3^, respectively. The measured and predicted density values are presented in Figure 4A. For most samples, the density was below 1.00 due to the ethanol concentration of the wines. On the other hand, wines with higher sugar levels can have a higher density than water. The density distribution of external test samples covers the applied range similarly as the training set samples.

For total alcohol concentration, 131 variables were selected by a genetic algorithm for modeling. The training set contained 299 samples, while the external set included 71 samples. Randomized fivefold cross-validation was used in the same way as detailed above. Twenty latent variables were used for the final PLS model. The permutation test verified that the model was not overfitted. The *R*^2^ values for the training set, CV and external test set were 0.92, 0.89 and 0.80, respectively. The root mean squared errors of the training, CV and external validation were 0.24, 0.30 and 0.39 *v*/*v*%, respectively. The predicted and measured values are plotted against each other in Figure 4B. The external test samples were well-distributed in the evaluated range of the alcohol concentration.

In the case of total sugar content, the number of measured samples was less compared to the others, but it was still sufficient for building a robust model. In total, 71 samples were used as the training set and 7 samples as the test set. The amount of external test samples was less than in the previous cases, but we had to preserve the stability of the model during training. A genetic algorithm has selected 259 variables out of 860 for modeling. The validation of the PLS model was done in the same way as previously, and the final model contained 10 latent variables. The permutation test showed that the model was not overfitted and significantly better than the one used with random numbers. The measured vs. predicted values are plotted in Figure 4C. The *R*^2^ values for the training, CV and external test set were 0.95, 0.88 and 0.95, respectively. The root mean squared errors for the training, CV and external test set were 4.99, 7.81 and 8.2 g/L, respectively. Most of the measured wine samples had a total sugar content level below 30 g/L which is reasonable, considering that there were more dry wines amongst the evaluated samples.

Our last PLS model was developed for the determination of SO_2_ concentration in the wine samples. It total, 250 samples were used for the training of the model, and the external test set contained 55 samples. The genetic algorithm selected 249 variables out of 860 for model building. The number of latent variables was 18. The appropriate robustness and reliability of the model was verified by fivefold randomized cross-validation with 20 iterations and permutation tests with 50 iterations. The *R*^2^ values for the training, CV and external test set were 0.88, 0.82 and 0.76, respectively. The root mean squared errors of training, CV and external test set prediction (RMSEC, RMSECV and RMSEP) were 12.60, 16.40 and 16.17 mg/L, respectively. Figure 4D shows the predicted and measured concentrations plotted against each other. It is not surprising, that the models performed slightly worse in external test validation, because SO_2_ is indirectly connected to ^1^H NMR spectra, based on its well-known effects on the organic metabolites in wines (polyphenols, enzyme reactivity, *etc.*) [30,31]. However, the model is still capable of predicting the SO_2_ concentration in wines with sufficient precision.

## 4. Conclusions

A comprehensive classification and regression analysis of Hungarian red and white wines have been carried out. In total, 403 wine samples were measured by ^1^H NMR spectroscopy, and the spectra were used for chemometric modeling. In the case of the classification of white wines, the sorts of Chardonnay, Sauvignon Blanc and Riesling were successfully predicted with the XGBoost algorithm combined with iterative double cross-validation and the performance of the prediction was above 0.8 for accuracy and above 0.9 for AUC values, even for the external test set. A similarly good classification resulted in the case of red wine varieties (Cabernet Franc, Merlot and Blue Frankish). The applied classification workflow can be applied in quality controls of wines.

As for the regression models, four major quantitative parameters of the wine samples were predicted: density, total sugar, total alcohol and total SO_2_ concentrations. The NMR spectra of the samples provided sufficient information to develop robust and reliable models with PLS regression. The goodness of the predictions (*R*^2^) was always above 0.80, except for the external test set of the SO_2_ concentration determination. All the four models are applicable for the substitution of the time-demanding reference measurement protocols. Moreover, the chemometric models based on ^1^H NMR data could provide additional information (qualitative and quantitative); thus, they are viable options for the replacement of the traditional standards either in the wine analysis or in other endeavors of food science.

## Figures and Tables

**Table 1:** Summary of the previous classification and regression studies based on nuclear magnetic resonance (NMR) spectra.

Type of Analysis	Method	Features	Number of Samples	Analytical Method	Reference
regression	PLS	ethanol, glycerol, lactic acid, methanol and malic acid	40	^1^H NMR	[14]
regression	Tchebichef moment-PLS	glycerol, ethanol, lactic acid, malic acid, methanol	40	^1^H NMR 3D spectra	[13]
classification	LDA, PLS-DA, FDA, ICA	geographical origin, red wine varieties, year of vintage	718	^1^H NMR and stable isotopes	[15]
classification	RF	white wine varieties	679	^1^H NMR	[16]
classification	LDA	wine varieties, geographical origin	107	^1^H NMR	[17]
classification	LDA, MANOVA	wine varieties, year of vintage, geographical origin	579	^1^H NMR	[18]
classification	LDA	wine varieties, year of vintage	56	HPLC, Isotopic analysis, ^1^H NMR, ^13^C NMR	[19]
classification	PCA, SOM, CA	addition of beet or cane sugar, geographical origin	50	SNIF-NMR, Isotopic ratio	[20]
classification	PLS-DA	wine varieties	58	HPLC, EEM, ^1^H NMR	[21]

LDA = linear discriminant analysis, PCA = principal component analysis, CA = cluster analysis, PLS/PLS-DA = partial least squares regression/discriminant analysis, RF = random forest, FDA = factorial discriminant analysis, ICA = independent component analysis, MANOVA = multiway analysis of variance, SOM = self-organizing maps (Kohonen networks), EEM = emission-excitation fluorescence spectroscopy.

**Table 2:** Number of samples used in the training and test sets in the regression models.

Type of Method	Model	Training Set	Test Set
PLS Regression	Density	278	71
	Total alcohol concentration	299	71
	Total sugar concentration	71	7
	Total SO_2_ concentration	250	55
Classification	White wine varieties	102	26
	Red wine varieties	97	25

**Table 3:** Summary of the performance parameters of the white and red wine classification.

Dataset	Validation	Accuracy	AUC
White	CV	0.833	0.952
	Internal test	0.814	0.948
	External test	0.808	0.922
Red	CV	0.814	0.920
	Internal test	0.804	0.921
	External test	0.800	0.965

## Data Availability

The data presented in this study are available on request from the corresponding author.

## Supplementary Materials

The following are available online at https://www.mdpi.com/2304-8158/10/1/64/s1, Figure S1: Pulse sequence for the ^1^H NMR measurements, Figure S2: Representative ^1^H NMR spectrum of a wine sample (after preprocessing), Figure S3: the applied KNIME workflow for the classification of the wine samples.
