# Supplementary material for: Comprehensive Classification and Regression Modeling of Wine Samples Using 1H NMR Spectra
Source: Foods. 2020 Dec 30;10(1):64. doi: 10.3390/foods10010064 (PMC7824661; doi:10.3390/foods10010064)

Figure S1. Pulse sequence for the $^{1}$H NMR measurments

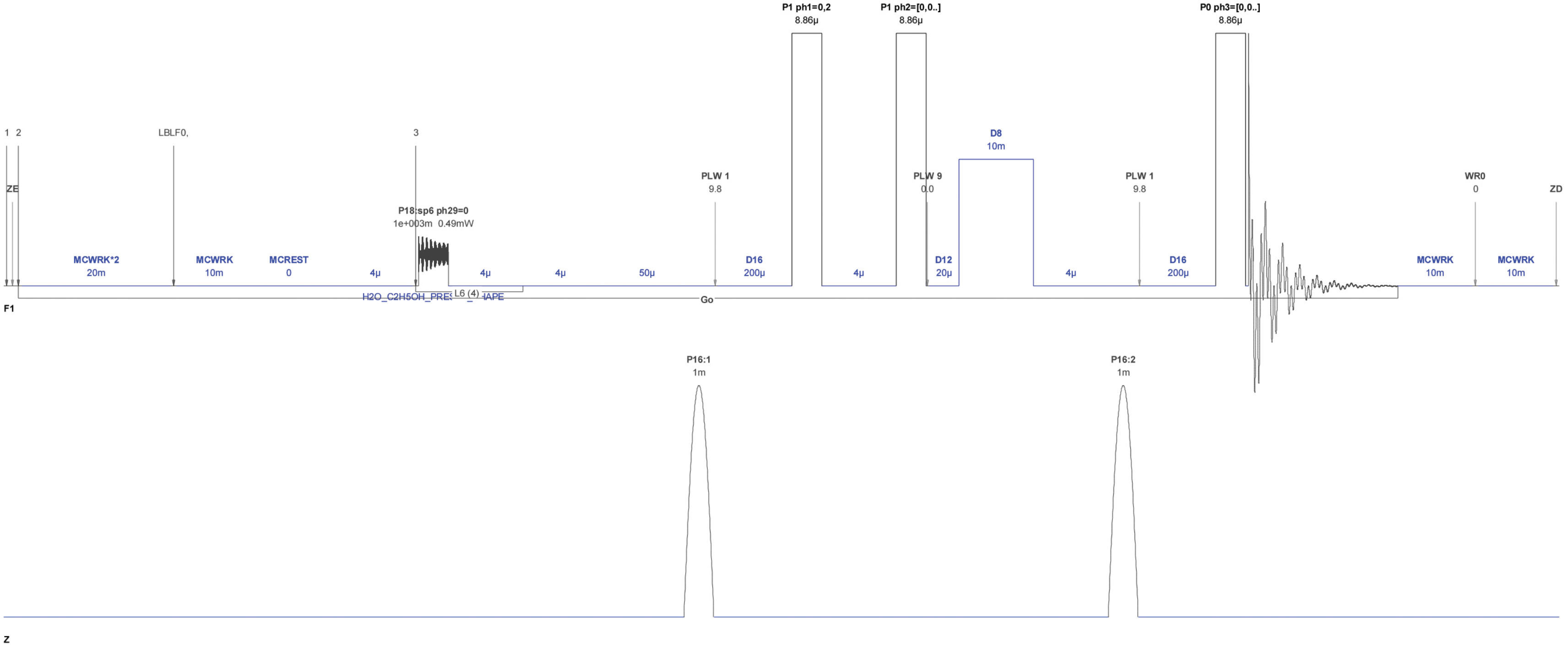

**Figure S2.** Representative $^{1}$H NMR spectrum of a wine sample (after preprocessing)

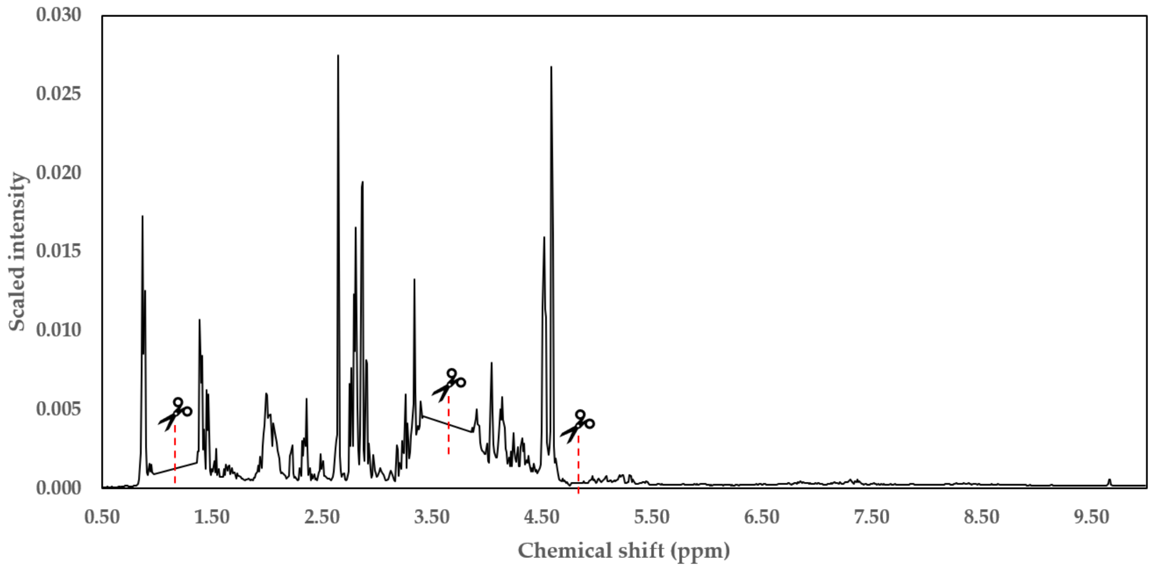

**Supplementary Figure S3:** the applied KNIME workflow for the classification of the wine samples

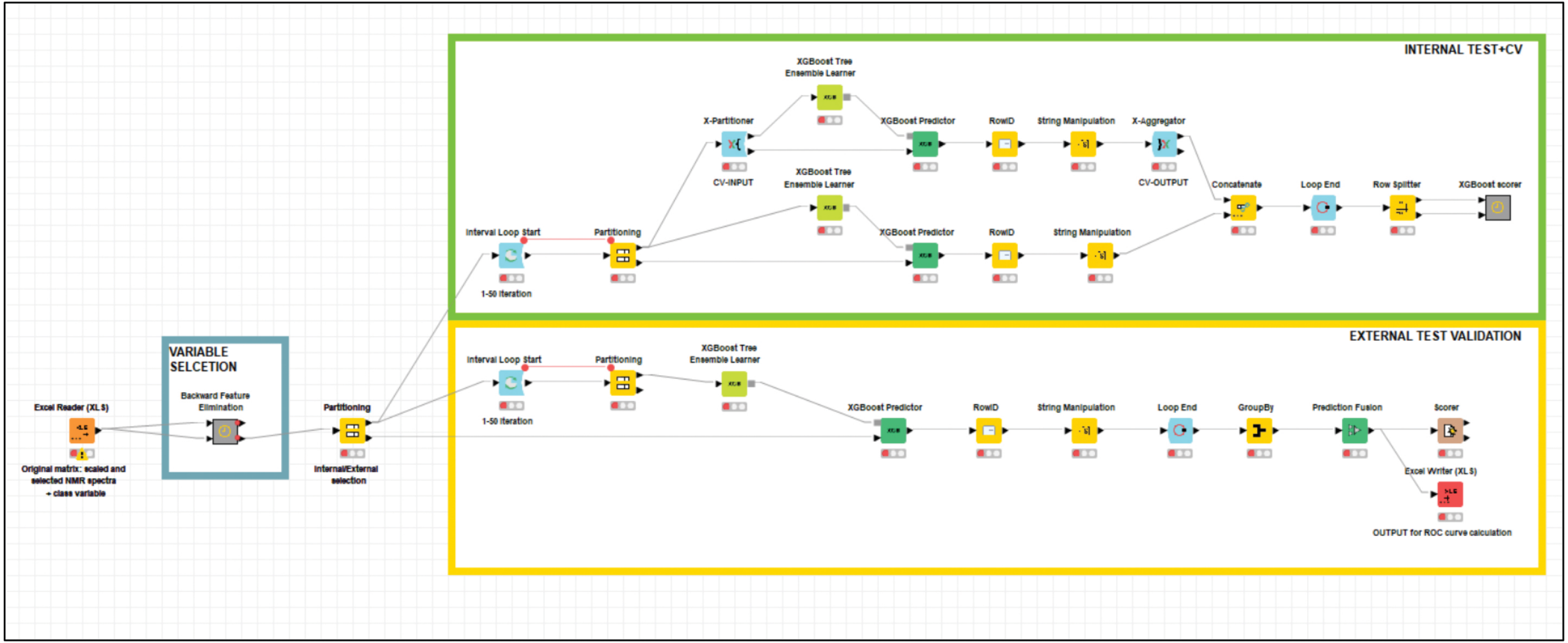

Supplement: Supplementary file 1 [file foods-10-00064-s001.pdf]
